# Supplementary material for: Cholesterol-rich lysosomes induced by respiratory syncytial virus promote viral replication by blocking autophagy flux
Source: Nat Commun. 2024 Jul 26;15:6311. doi: 10.1038/s41467-024-50711-4 (PMC11282085; doi:10.1038/s41467-024-50711-4)
Supplement: Supplementary file 1 — Supplementary Information [file 41467_2024_50711_MOESM1_ESM.pdf]

Supplementary information

Cholesterol-rich lysosomes induced by respiratory syncytial virus promote viral replication by blocking autophagy flux

Supplementary Table 1 siRNA target sequence

siRNA	Target sequence（5'-3'）
si-ORP1L	GCCGGATTCTGAAAGTGTA
si-LDLR	CGGAGAAGCTGCCTATCAA

Supplementary Table 2 Primer sequences

Genes	Forward primer (5′–3′)	Reverse primer (5′–3′)
LAL	TTACAACCAGAGTTATCCTCCCA	CCAAATGAAGTCAAGATGCTCCC
LDLR	CTGGAGGGTGGCTACAAGTGC	GCCGGTTGGTGAAGAAGAGGT
HMGCR	TTCGCAGTGATAAAGGAGGCA	CCTGACCTGGACTGGAAACG
RSV NS1	TGTATGTATCACTGCCTTAGCCAAAG	ATGGGCAGCAATTCATTGAGTATG
RSV NS2	TGGGAGTATGCTTTGTAGGCTTAAT	GCCACATTTACATTCCTGGTCAA
RSV F	GAGGGGAATACTAATGGGTCATAGAA	TCAAATAAAGGGGTGGACACTGT
Human-GAPDH	TGATGACCCTTTTGGCTCCC	AAATCCCATCACCATCTTCCAG
Mouse-GAPDH	AGGTCGGTGTGAACGGATTTG	TGTAGACCATGTAGTTGAGGTCA

Results

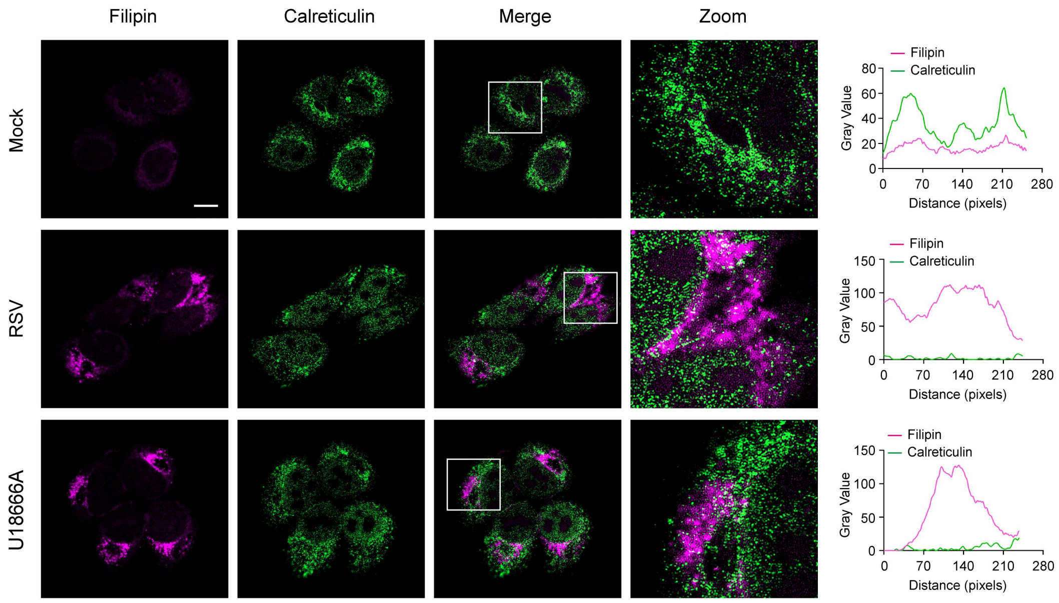

Supplementary Figure 1. RSV infection blocks cholesterol transport to the ER in infected cells. HEp-2 cells were either mock-infected or infected with RSV (MOI = 1) in the presence or absence of U18666A (10 μM) for 24 h. Immunocolocalization of cholesterol (filipin III) and calreticulin in mock-infected, RSV-infected (24 h post-infection), and U18666A-treated HEp-2 cells. Scale bar: 10 μm. Data are one representative of three independent experiments. Image parameters: Scaling-per Pixel (0.032 μm × 0.032 μm); Image Size-Pixels (2432 × 2432); Image Size-Scaled (78.01 μm × 78.01 μm); Objective (Plan-Apochromat 63×/1.40 Oil DIC M27); Scan Zoom (1.3).

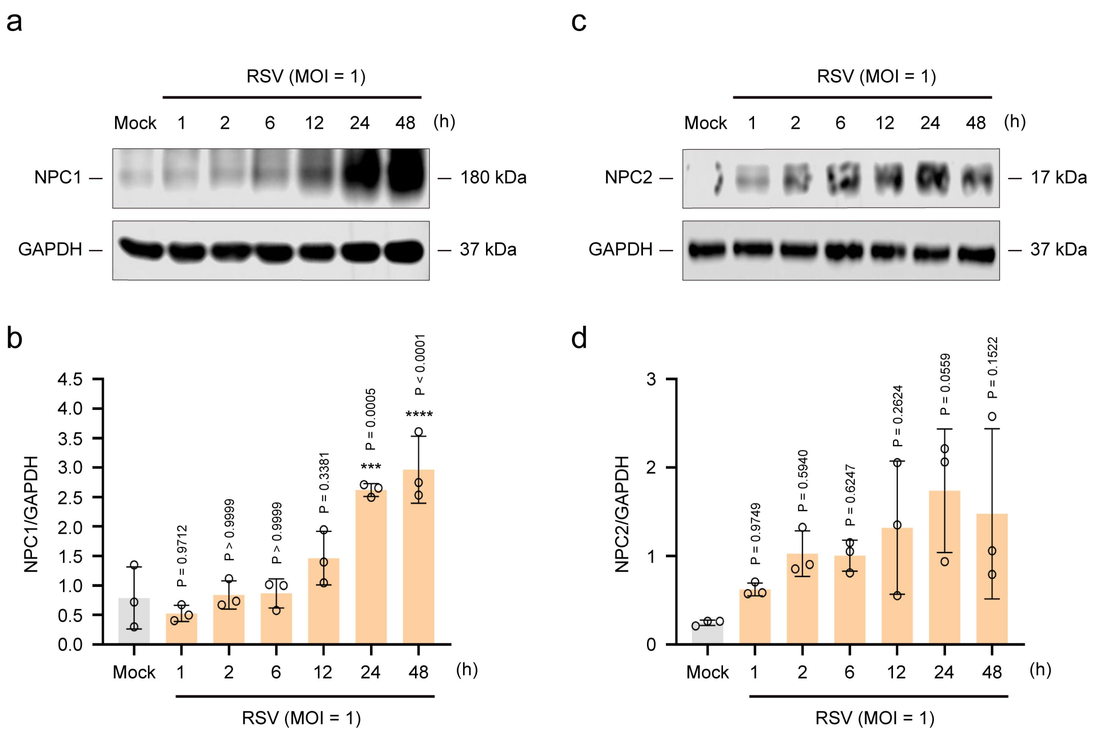

Supplementary Figure 2. RSV infection increases the expression of NPC1 and NPC2 in infected cells. HEp-2 cells were either mock-infected or infected with RSV (MOI = 1) for 0, 1, 2, 6, 12, 24, or 48 h. a–d The protein levels of NPC1 and NPC2 were determined using western blotting (n = 3 independent experiments). Data are shown as the mean ± SD, statistical analysis using one-way ANOVA (***$P < 0.001$ and ****$P < 0.0001$ compared to the blank control group).

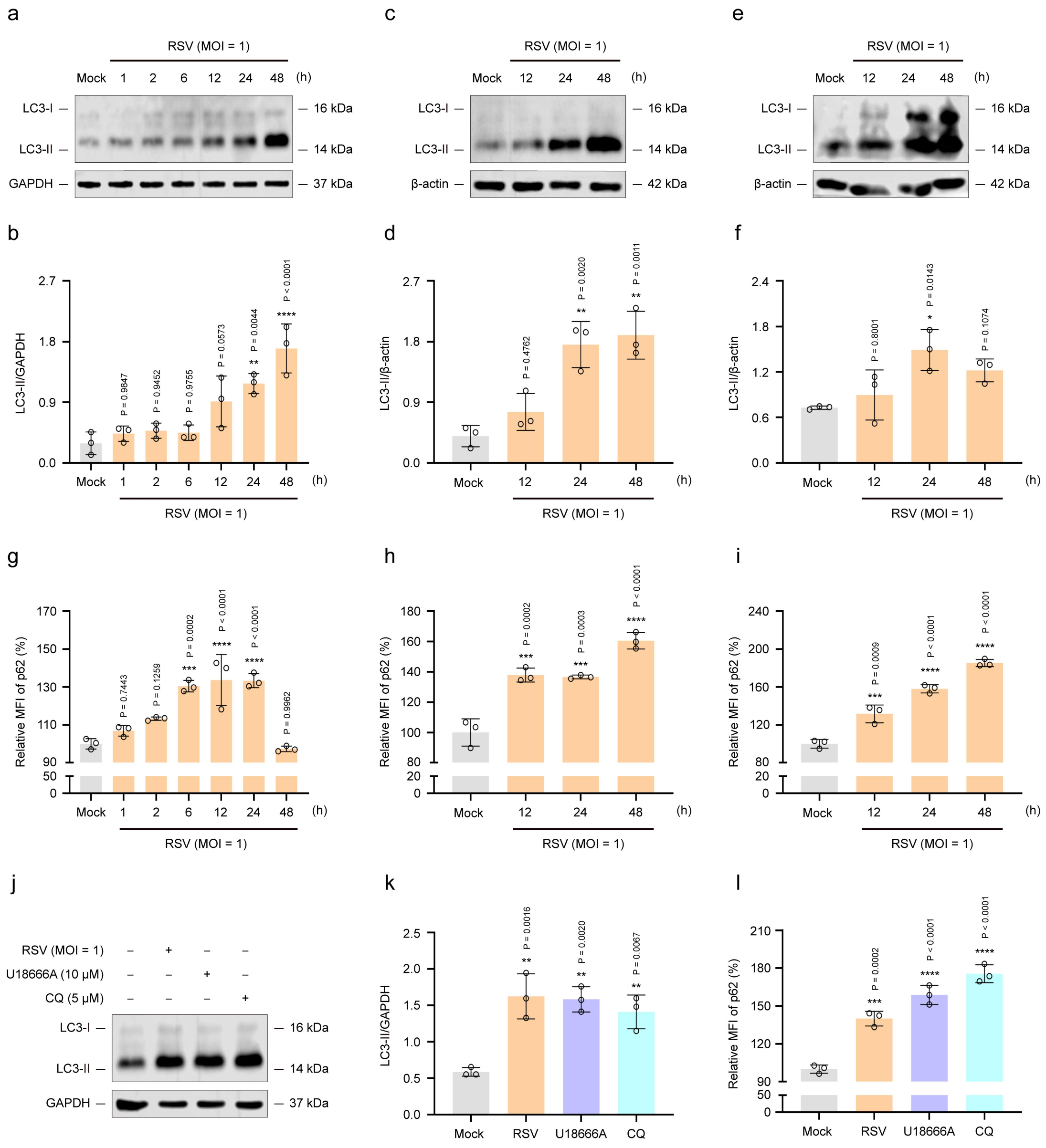

Supplementary Figure 3. RSV infection inhibits autophagy flux in infected cells. HEp-2, 16HBE, or HBECs cells were either mock-infected or infected with RSV (MOI = 1) in the presence or absence of U18666A (10 μM) or CQ (5 μM) for the indicated durations. a–f, j, k The protein level of LC3B was determined using western blotting (HEp-2: a, b, j, k; 16HBE: c, d; HBECs: e, f) (n = 3 independent experiments). g–i, l The protein level of p62 was determined using an immunofluorescence assay (HEp-2: g, l; 16HBE: h; HBECs: i) (n = 3 independent experiments). Data are shown

as the mean ± SD, statistical analysis using one-way ANOVA (*P < 0.05, **P < 0.01, ***P < 0.001, and ****P < 0.0001 compared to the blank control group).

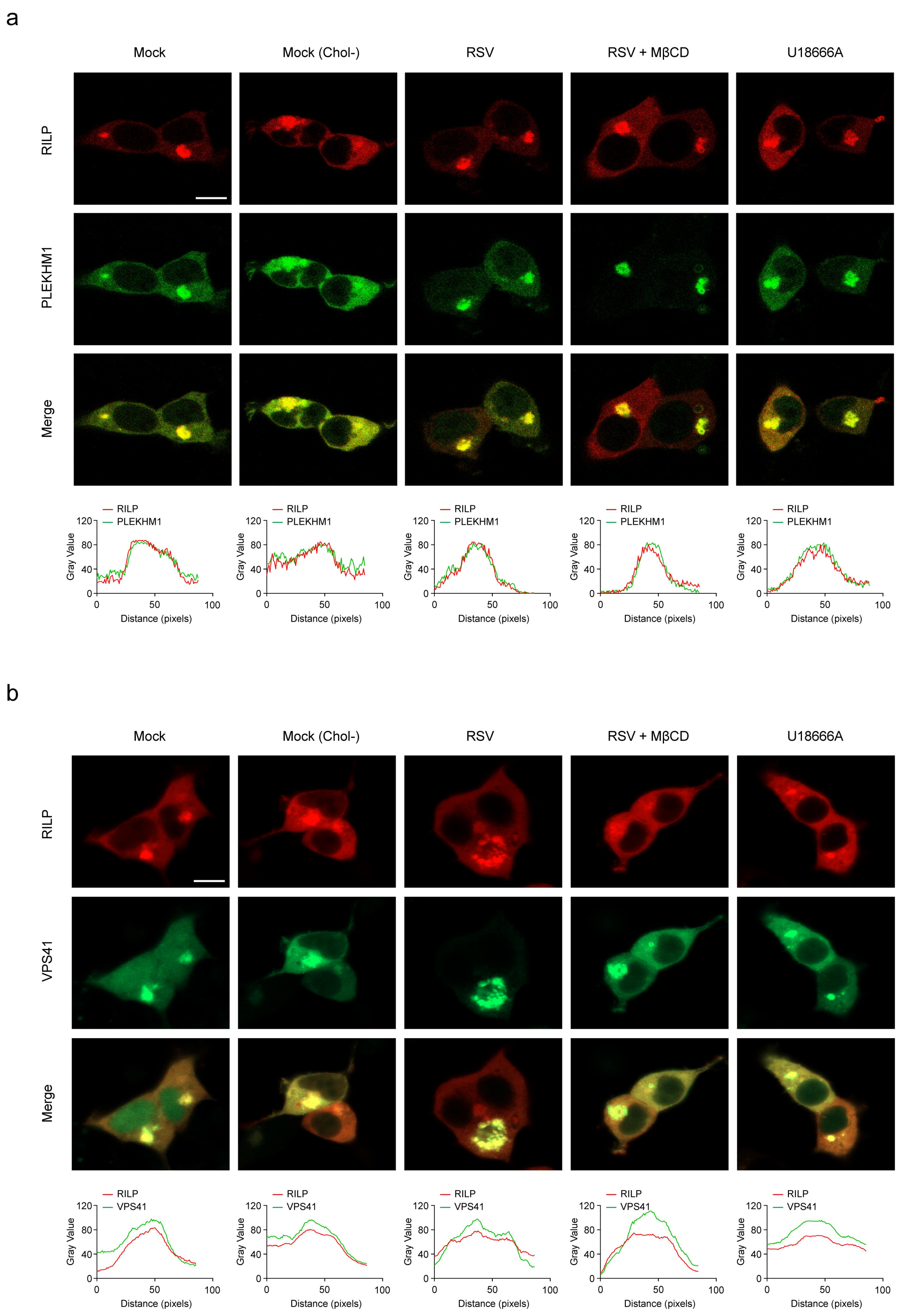

Supplementary Figure 4. RSV infection does not affect the interaction between RILP and PLEKHM1/HOPS VPS41 in infected cells. HEK293T cells transiently expressing the indicated plasmids were either mock-infected or infected with RSV

(MOI = 1) in the presence or absence of U18666A (10 μM) or MβCD (100 μM) for 24 h. a, b The interaction between RILP and PLEKHM1/HOPS VPS41 was determined using an immunofluorescence assay. Scale bar: 10 μm. Data are one representative of three independent experiments.

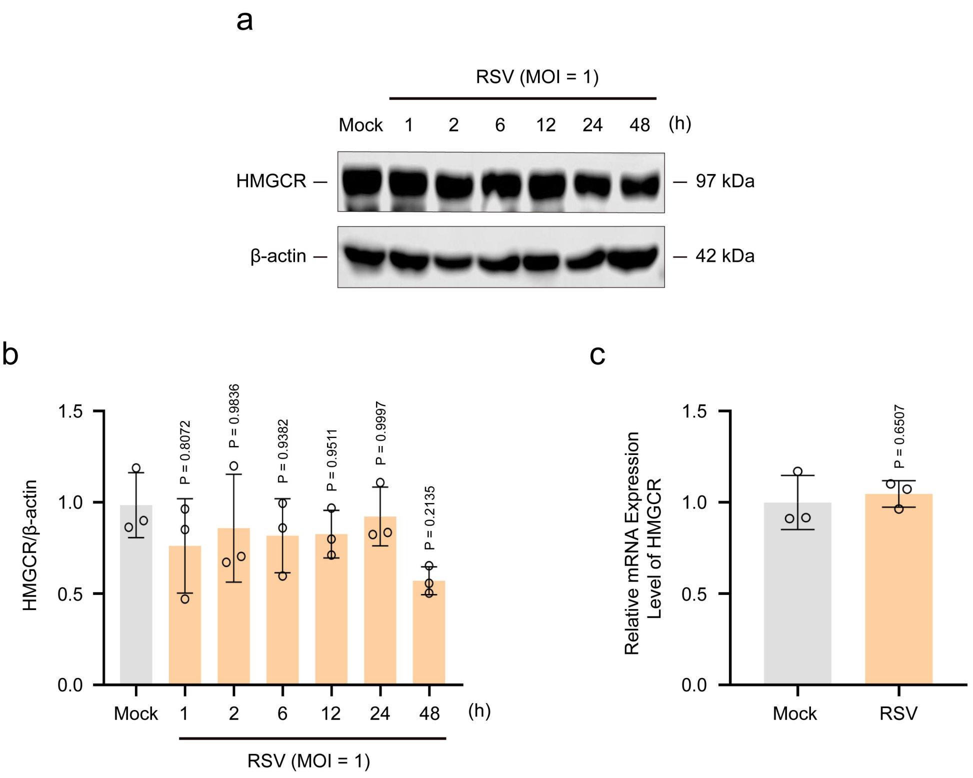

Supplementary Figure 5. RSV infection does not affect HMGCR expression in infected cells. HEp-2 cells were either mock-infected or infected with RSV (MOI = 1) for the indicated durations. a, b The protein level of HMGCR 0, 1, 2, 6, 12, 24, or 48 h after RSV infection was determined using western blotting (n = 3 independent experiments). c The mRNA level of *HMGCR* gene 24 h after RSV infection was determined using RT-PCR (n = 3 independent experiments). Data are shown as the

mean ± SD, statistical analysis using two-sided Student's t-test (c) or one-way ANOVA (b).

Fig. 2a

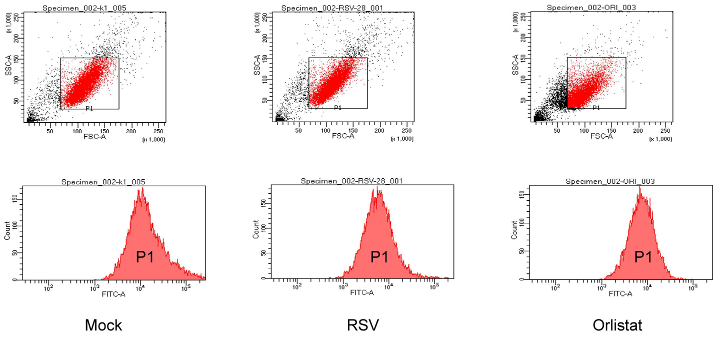

Fig. 3h

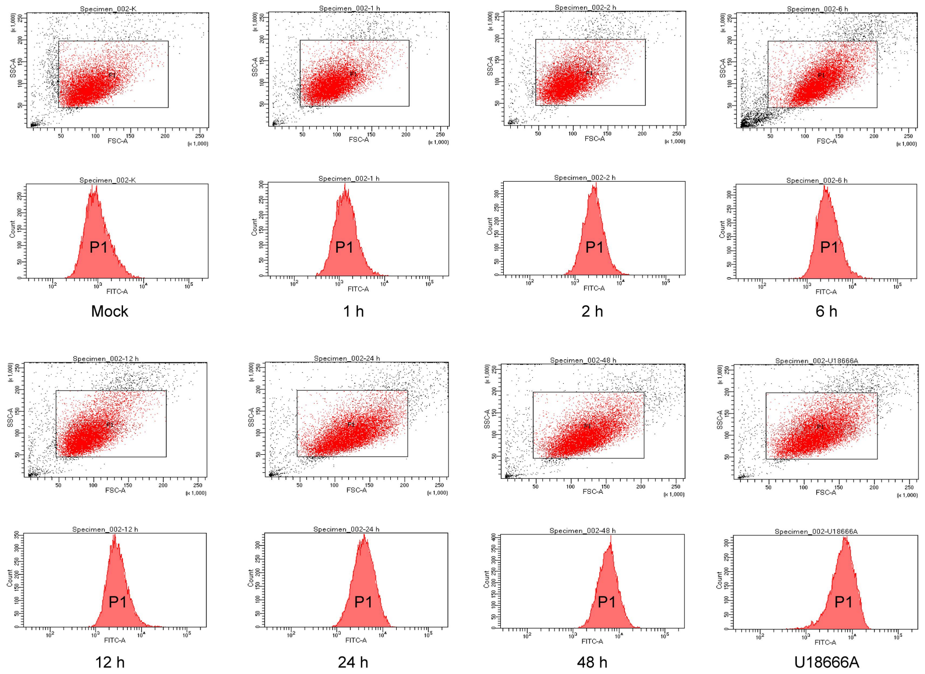

Fig. 6h

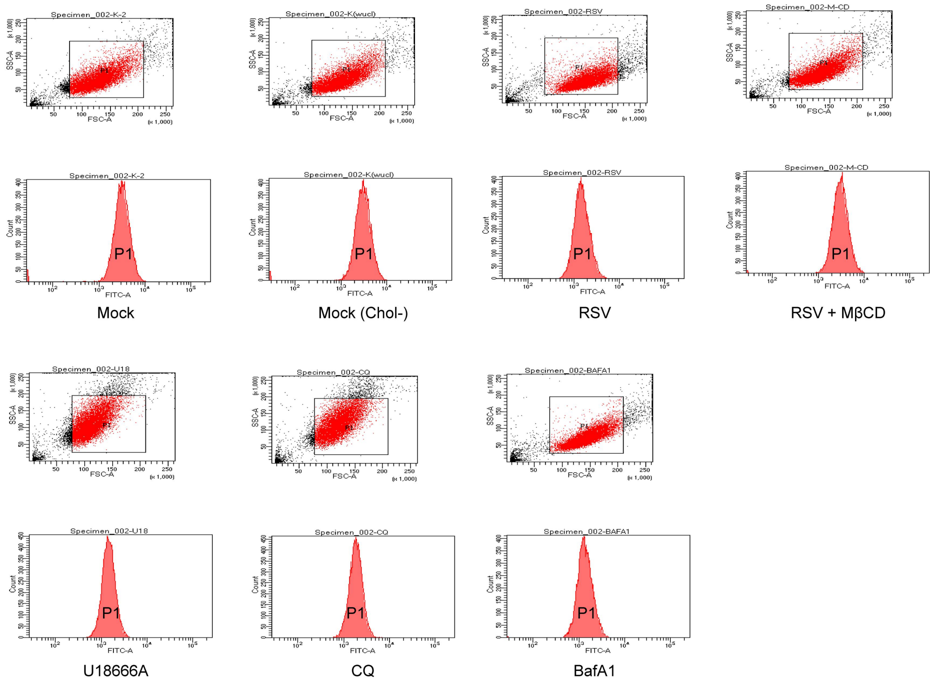

FACS data
