# Supplementary material for: Cholesterol-rich lysosomes induced by respiratory syncytial virus promote viral replication by blocking autophagy flux
Source: Nat Commun. 2024 Jul 26;15:6311. doi: 10.1038/s41467-024-50711-4 (PMC11282085; doi:10.1038/s41467-024-50711-4)
Supplement: Supplementary file 3 — Reporting Summary [file 41467_2024_50711_MOESM3_ESM.pdf]

nature portfolio

Corresponding author(s): Professor Zhong Liu

Last updated by author(s): June 21st, 2024

Reporting Summary

Nature Portfolio wishes to improve the reproducibility of the work that we publish. This form provides structure for consistency and transparency in reporting. For further information on Nature Portfolio policies, see our Editorial Policies and the Editorial Policy Checklist.

Statistics

For all statistical analyses, confirm that the following items are present in the figure legend, table legend, main text, or Methods section.

n/a	Confirmed	
☐	☒	The exact sample size (*n*) for each experimental group/condition, given as a discrete number and unit of measurement
☐	☒	A statement on whether measurements were taken from distinct samples or whether the same sample was measured repeatedly
☐	☒	The statistical test(s) used AND whether they are one- or two-sided<br>*Only common tests should be described solely by name; describe more complex techniques in the Methods section.*
☒	☐	A description of all covariates tested
☐	☒	A description of any assumptions or corrections, such as tests of normality and adjustment for multiple comparisons
☐	☒	A full description of the statistical parameters including central tendency (e.g. means) or other basic estimates (e.g. regression coefficient) AND variation (e.g. standard deviation) or associated estimates of uncertainty (e.g. confidence intervals)
☐	☒	For null hypothesis testing, the test statistic (e.g. *F*, *t*, *r*) with confidence intervals, effect sizes, degrees of freedom and *P* value noted<br>*Give P values as exact values whenever suitable.*
☒	☐	For Bayesian analysis, information on the choice of priors and Markov chain Monte Carlo settings
☒	☐	For hierarchical and complex designs, identification of the appropriate level for tests and full reporting of outcomes
☒	☐	Estimates of effect sizes (e.g. Cohen's *d*, Pearson's *r*), indicating how they were calculated

Our web collection on statistics for biologists contains articles on many of the points above.

Software and code

Policy information about availability of computer code

Data collection

1. Amersham Imager 600 (AI600)
2. Carl Zeiss AG (LSM800)
3. Multimodality Structured Illumination Microscopy X
4. Abberior STEDYCON
5. BD Biosciences (BD FACSCanto)
6. Hitachi H-7500 (HITACHI)
7. Microplate reader (Synergy H1)
8. Quantitative PCR system (LightCycler 480)
9. Five-part differential automated hematological analyzer (HEVMAVET 950)
10. Optical microscope (BX53F2)

Data analysis	1. ImageJ V1.6.0<br>2. GraphPad Prism V.9.4.0<br>3. FlowJo V10<br>4. WPS 12.1.0.17133<br>5. Imaris 10.1

For manuscripts utilizing custom algorithms or software that are central to the research but not yet described in published literature, software must be made available to editors and reviewers. We strongly encourage code deposition in a community repository (e.g. GitHub). See the Nature Portfolio guidelines for submitting code & software for further information.

Data

Policy information about availability of data

All manuscripts must include a data availability statement. This statement should provide the following information, where applicable:

- Accession codes, unique identifiers, or web links for publicly available datasets
- A description of any restrictions on data availability
- For clinical datasets or third party data, please ensure that the statement adheres to our policy

All source data are provided with this paper.

Research involving human participants, their data, or biological material

Policy information about studies with human participants or human data. See also policy information about sex, gender (identity/presentation), and sexual orientation and race, ethnicity and racism.

Reporting on sex and gender	The study did not involve this item.
Reporting on race, ethnicity, or other socially relevant groupings	The study did not involve this item.
Population characteristics	The study did not involve this item.
Recruitment	The study did not involve this item.
Ethics oversight	The study did not involve this item.

Note that full information on the approval of the study protocol must also be provided in the manuscript.

Field-specific reporting

Please select the one below that is the best fit for your research. If you are not sure, read the appropriate sections before making your selection.

[x] Life sciences   [ ] Behavioural & social sciences   [ ] Ecological, evolutionary & environmental sciences

For a reference copy of the document with all sections, see nature.com/documents/nr-reporting-summary-flat.pdf

Life sciences study design

All studies must disclose on these points even when the disclosure is negative.

Sample size	No statistical method was used to predetermine the sample size. For in vitro studies, the experiment was repeated at least 3 times with a sample size of at least 3 to control for technical changes. For in vivo studies, six animals were used in each group to obtain useful results.
Data exclusions	No data were excluded from the analyses.
Replication	All experiments were repeated at least three times, and all attempts at replication were successful.
Randomization	Random.
Blinding	No blinding was used in this study. All data collected was quantifiable and blinding would not change any bias in data collected.

Reporting for specific materials, systems and methods

We require information from authors about some types of materials, experimental systems and methods used in many studies. Here, indicate whether each material, system or method listed is relevant to your study. If you are not sure if a list item applies to your research, read the appropriate section before selecting a response.

Materials & experimental systems

n/a	Involved in the study	
	☒	Antibodies
	☒	Eukaryotic cell lines
☒		Palaeontology and archaeology
	☒	Animals and other organisms
☒		Clinical data
☒		Dual use research of concern
☒		Plants

Methods

n/a	Involved in the study	
☒		ChIP-seq
	☒	Flow cytometry
☒		MRI-based neuroimaging

Antibodies

Antibodies used

The following antibodies were used for immunofluorescence assay (IF) or western blotting (WB);
1. Anti-Respiratory Syncytial Virus Fusion (F) Glycoprotein (abcam, catalogue no.ab94968, IF 1:1000 dillution, LOT: GR3370956-1)
2. Anti-Respiratory Syncytial Virus G Glycoprotein (abcam, catalogue no.ab94966, IF 1:1000 dillution, LOT: GR3247185-12)
3. Anti-Respiratory Syncytial Virus Nucleoprotein (abcam, catalogue no.ab94806, IF 1:1000 dillution, LOT: 1029087-8)
4. Anti-beta Actin antibody [mAbcam 8226]-Loading Control (abcam, catalogue no.ab8226, WB 1:1000 dillution, LOT: GR3396181-2)
5. Goat Anti-Mouse IgG H&L (Alexa Fluor® 488) (abcam, catalogue no.ab150113, IF 1:1000 dillution, LOT: GR3419505-1)
6. Goat Anti-Rabbit IgG H&L (Alexa Fluor® 488) (abcam, catalogue no.ab150077, IF 1:1000 dillution, LOT: 1052444-22)
7. Goat Anti-Rabbit IgG H&L (Alexa Fluor® 594) (abcam, catalogue no.ab150080, IF 1:1000 dillution, LOT: GR3373513-1)
8. Goat Anti-Mouse IgG H&L (Alexa Fluor® 594) (abcam, catalogue no.ab150116, IF 1:1000 dillution, LOT: GR3438808-1)
9. Goat Anti-Rabbit IgG H&L (Alexa Fluor® 647) (abcam, catalogue no.ab150079, IF 1:1000 dillution, LOT: 3444080-2)
10. Anti-ORP1 antibody [EPR8646] (abcam, catalogue no.ab131165, WB 1: 1000 dillution, LOT: GR3283699-8)
11. Anti-LDL Receptor antibody [EP1553Y] (abcam, catalogue no.ab52818, WB 1:1000 dillution, LOT: 1000292-1)
12. Anti-HMGCR antibody [CL0260] (abcam, catalogue no.ab242315, WB 1:1000 dillution, LOT: 1037157-2)
13. Anti-Niemann Pick C1 antibody [EPR5209] (abcam, catalogue no.ab134113, WB 1:2000 dillution, LOT: GR3230423-6)
14. Anti-Niemann Pick C2 antibody [EPR19993-145-1] (abcam, catalogue no.ab218192, WB 1:2000 dillution, LOT: GR3421834-3)
15. Anti-Calreticulin antibody [EPR3924]-ER Marker (abcam, catalogue no.ab92516, IF 1:500 dillution, LOT: GR3287998-11)
16. Anti-rabbit IgG, HRP-linked Antibody (Cell Signaling Technology, catalogue no.7074, WB 1:2000 dillution, LOT: 32)
17. Anti-mouse IgG, HRP-linked Antibody (Cell Signaling Technology, catalogue no.7076, WB 1:2000 dillution, LOT: 38)
18. LAMP1 (D2D11) XP® Rabbit mAb (Cell Signaling Technology, catalogue no.9091S, IF 1:400 dillution, LOT: 6)
19. LC3B (E5Q2K) Mouse mAb (Cell Signaling Technology, catalogue no.83506S, WB 1:1000 dillution, IF 1:800 dillution, LOT: 2)
20. LAMP1 (D4O1S) Mouse mAb (Cell Signaling Technology, catalogue no.15665S, IF 1:100 dillution, LOT: 4)
21. SREBP-2 Antibody (1C6) (Santacruz Biotechnology, catalogue no.sc-13552, WB 1:1000 dillution, IF 1:500 dillution, LOT: GR3376305-1)
22. LAL Antibody (Affinity, catalogue no.BF0079, WB 1:1000 dillution, LOT:1 9c1337)
23. Anti-p62 (SQSTM1) pAb (MBL, catalogue no.PM045, IF 1:400 dillution, LOT: 021)
24. GAPDH Rabbit Recombinant mAb (Bimake, catalogue no.A5028, WB 1:1000 dillution, LOT: GR3401390-1)
25. Anti-SQSTM1/p62 pAb (abcam, catalogue no.ab109012, IF 1:400 dillution, LOT: 1000346-40)

Validation

All antibodies sourced from commercial corporation are well-validated by the manufacturer. The following antibodies were used for immunofluorescence assay (IF) and western blotting (WB);
1. https://www.abcam.cn/products/primary-antibodies/respiratory-syncytial-virus-fusion-f-glycoprotein-antibody-rsv5a6-ab94968.html
2. https://www.abcam.cn/products/primary-antibodies/respiratory-syncytial-virus-g-glycoprotein-antibody-rsv133-ab94966.html
3. https://www.abcam.cn/products/primary-antibodies/respiratory-syncytial-virus-nucleoprotein-antibody-rsv1c3-ab94806.html
4. https://www.abcam.cn/products/primary-antibodies/beta-actin-antibody-mabcam-8226-loading-control-ab8226.html
5. https://www.abcam.cn/products/secondary-antibodies/goat-mouse-igg-hl-alexa-fluor-488-ab150113.html
6. https://www.abcam.cn/products/secondary-antibodies/goat-rabbit-igg-hl-alexa-fluor-488-ab150077.html
7. https://www.abcam.cn/products/secondary-antibodies/goat-rabbit-igg-hl-alexa-fluor-594-ab150080.html
8. https://www.abcam.cn/products/secondary-antibodies/goat-mouse-igg-hl-alexa-fluor-594-ab150116.html
9. https://www.abcam.cn/products/secondary-antibodies/goat-rabbit-igg-hl-alexa-fluor-647-ab150079.html
10. https://www.abcam.cn/products/primary-antibodies/orp1-antibody-epr8646-ab131165.html
11. https://www.abcam.cn/products/primary-antibodies/ldl-receptor-antibody-ep1553y-ab52818.html
12. https://www.abcam.cn/products/primary-antibodies/hmgcr-antibody-cl0260-ab242315.html
13. https://www.abcam.cn/products/primary-antibodies/niemann-pick-c1-antibody-epr5209-ab134113.html
14. https://www.abcam.cn/products/primary-antibodies/niemann-pick-c2-antibody-epr19993-145-1-ab218192.html
15. https://www.abcam.cn/products/primary-antibodies/calreticulin-antibody-epr3924-er-marker-ab92516.html
16. https://www.cellsignal.cn/products/secondary-antibodies/anti-rabbit-igg-hrp-linked-antibody/7074
17. https://www.cellsignal.cn/products/secondary-antibodies/anti-mouse-igg-hrp-linked-antibody/7076
18. https://www.cellsignal.cn/products/primary-antibodies/lamp1-d2d11-xp-rabbit-mab/9091
19. https://www.cellsignal.cn/products/primary-antibodies/lc3b-e5q2k-mouse-mab/83506
20. https://www.cellsignal.cn/products/primary-antibodies/lamp1-d4o1s-mouse-mab/15665

21. https://www.scbt.com/p/srebp-2-antibody-1c6?requestFrom=search
22. https://www.affbiotech.cn/goods-38-BF0079-LAL_Antibody.html
23. https://www.mbl-chinawide.cn/search012?keyword=PM045
24. https://www.selleck.cn/antibodies/gapdh-rabbit-recombinant-mab.html
25. https://www.abcam.cn/products/primary-antibodies/sqstm1--p62-antibody-epr4844-autophagosome-marker-ab109012.html

Eukaryotic cell lines

Policy information about cell lines and Sex and Gender in Research

Cell line source(s)	HEp-2 (ATCC CCL-23), 16HBE (ATCC CRL-9609), and HEK293T cells (ATCC CRL-3216) were purchased from the American Type Culture Collection (ATCC). HBECs cells (IMP-H041) were purchased from IMMOCELL.
Authentication	The cell lines were authenticated by the providers through morphology, karyotyping and PCR-based approaches. After receipt of the cell lines, visual inspection of their cellular morphology in culture was routinely performed and no further authentication was conducted in our lab.
Mycoplasma contamination	All cell lines tested negative for mycoplasma contamination.
Commonly misidentified lines (See ICLAC register)	No commonly misidentified cell lines were used in this study.

Animals and other research organisms

Policy information about studies involving animals; ARRIVE guidelines recommended for reporting animal research, and Sex and Gender in Research

Laboratory animals	Three-week-old male C57BL/6 mice (WT) or LDLR-/- C57BL/6 mice (Genetic background: 000664 C57BL/6J) were purchased from Cavens and maintained in a pathogen-free room (a 12 h light/dark cycle from 6:00 am to 6:00 pm and temperature of 22 ± 2 °C with 40–70% humidity) with free access to water and food.
Wild animals	This study did not involve wild animals.
Reporting on sex	Three-week-old male C57BL/6 mice (WT) or LDLR-/- C57BL/6 mice.
Field-collected samples	This study did not involve field-collected samples.
Ethics oversight	All mice were handled in strict adherence to the Guidelines for Laboratory Animal Use and Care of the Chinese Center for Disease Control and Prevention (CDC) and the Rules for Medical Laboratory Animals of the Ministry of Health, China. The animal study protocol was approved by the Ethics Committee of Jinan University and the National Institute for Communicable Disease Control and Prevention. The institution reference ID for animal ethics approval is 20220314-14.

Note that full information on the approval of the study protocol must also be provided in the manuscript.

Plants

Seed stocks	The study did not involve this item.
Novel plant genotypes	The study did not involve this item.
Authentication	The study did not involve this item.

Flow Cytometry

Plots

Confirm that:

- [x] The axis labels state the marker and fluorochrome used (e.g. CD4-FITC).
- [x] The axis scales are clearly visible. Include numbers along axes only for bottom left plot of group (a 'group' is an analysis of identical markers).
- [x] All plots are contour plots with outliers or pseudocolor plots.
- [x] A numerical value for number of cells or percentage (with statistics) is provided.

Methodology

Sample preparation	LAL assay. LAL activity was determined using a LysoLive™ Lysosomal Acid Lipase Assay Kit (Abcam) according to the manufacturer's instructions. Briefly, HEp-2 cells were mock-infected or infected with RSV (MOI = 1) for 24 h. Orlistat (10 μM) was used as a control. The monolayer was then treated with LysoLive™ LipaGreen™ (1 μL per well) and incubated at 37 °C for 4 h. After resuspending cells with 500 μL 1× flow holding and sorting buffer, the fluorescence signal of LipaGreen™ was detected on a flow cytometer using the FITC channel (BD Biosciences).<br>Dil-LDL uptake assay. HEp-2 cells were mock-infected or infected with RSV (MOI = 1) for 0, 1, 2, 6, 12, 24, or 48 h. U18666A (10 μM) was used as a control. The monolayer was then treated with Dil-LDL (30 μg/mL; Solarbio Science & Technology Co.,Ltd., Beijing, China) and incubated at 37 °C for 30 min. After washing twice with PBS, the cells were collected, and the fluorescence signal of Dil-LDL was detected on a flow cytometer using the FITC channel (BD Biosciences).<br>Lysosomal intracellular activity assay. Lysosomal activity was determined using a Lysosomal Intracellular Activity Assay Kit (Abcam) according to the manufacturer's instructions. Briefly, HEp-2 cells were mock-infected or infected with RSV (MOI = 1) in the presence or absence of cholesterol or MβCD (100 μM) for 24 h. U18666A (10 μM), CQ (5 μM), and BafA1 (200 nM) were used as controls. The monolayers were then treated with self-quenched substrate (15 μL per well) and incubated at 37 ° C for 1 h. After washing the cells twice with 1 mL ice-cold 1× assay buffer, the fluorescence signal of the self-quenched substrate was detected on a flow cytometer using the FITC channel (BD Biosciences).
Instrument	BD Biosciences
Software	BD FACSDiva and FlowJo V10 were used to collect and analyze the flow cytometric data.
Cell population abundance	10000 cells per tube were collected.
Gating strategy	All cells were gated based on size and granularity using FSC-A vs SSC-A. FITC positive cells were identified by comparing with the control samples.

- [x] Tick this box to confirm that a figure exemplifying the gating strategy is provided in the Supplementary Information.
