# Supplementary material for: Cholesterol-rich lysosomes induced by respiratory syncytial virus promote viral replication by blocking autophagy flux
Source: Nat Commun. 2024 Jul 26;15:6311. doi: 10.1038/s41467-024-50711-4 (PMC11282085; doi:10.1038/s41467-024-50711-4)

Figure 1

1g

HEp-2 HBECs

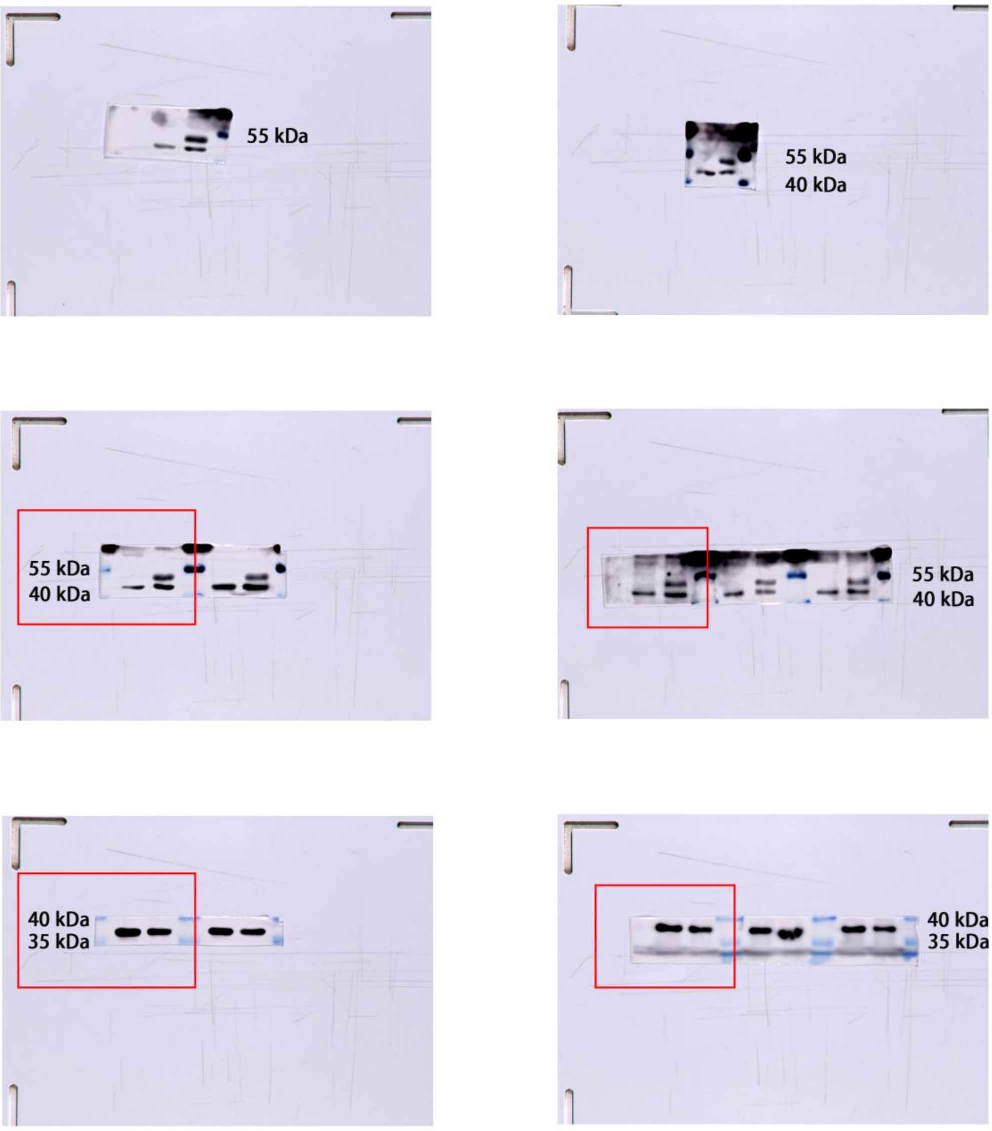

Figure 2

2d

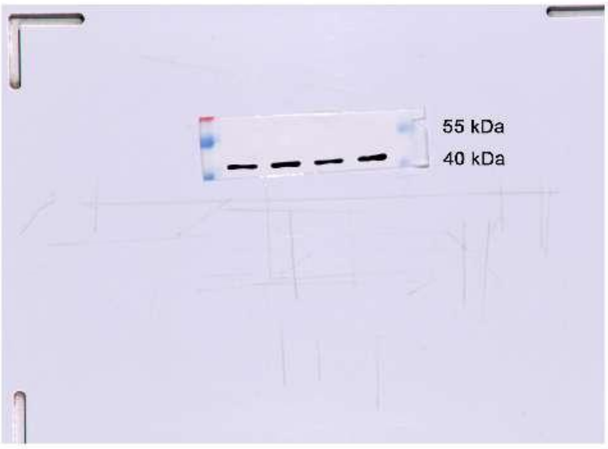

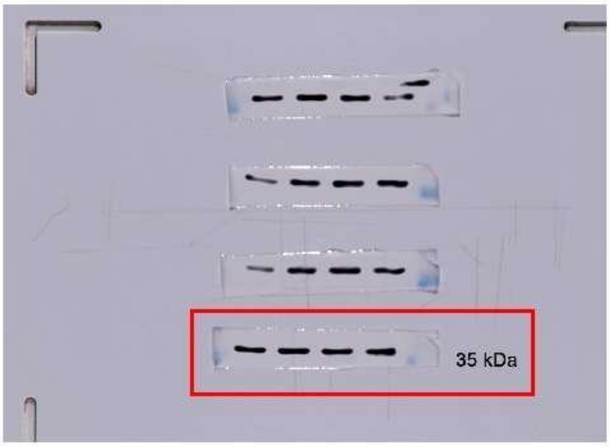

Figure 3

3a

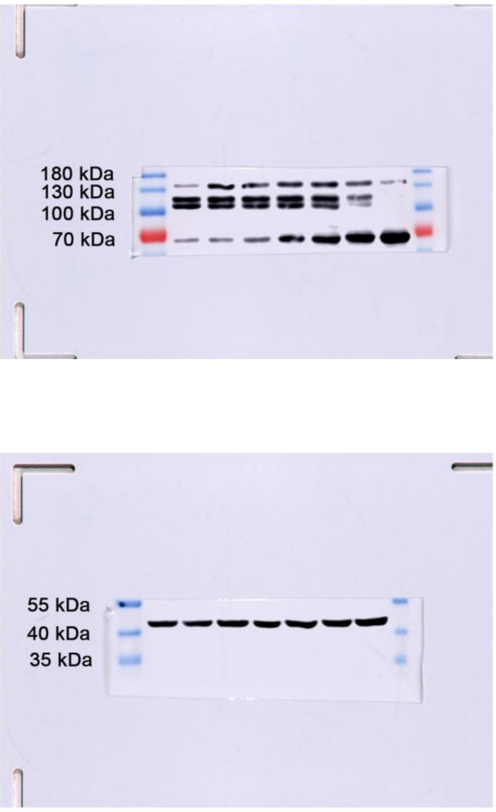

3e

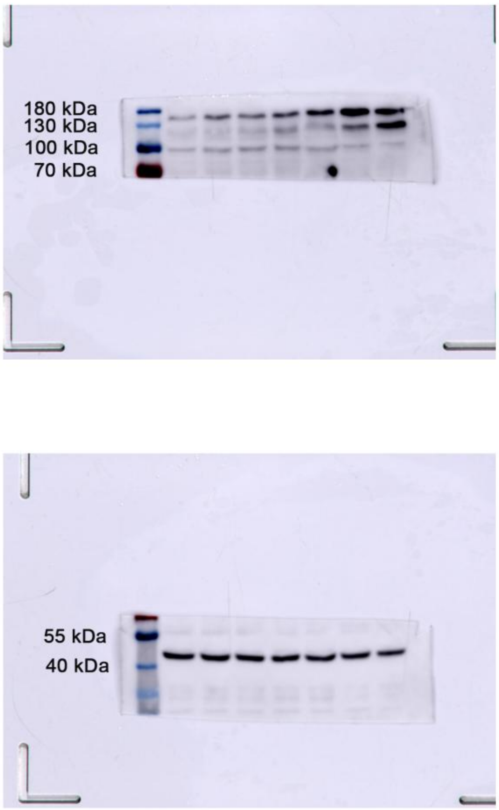

Figure 4

4h

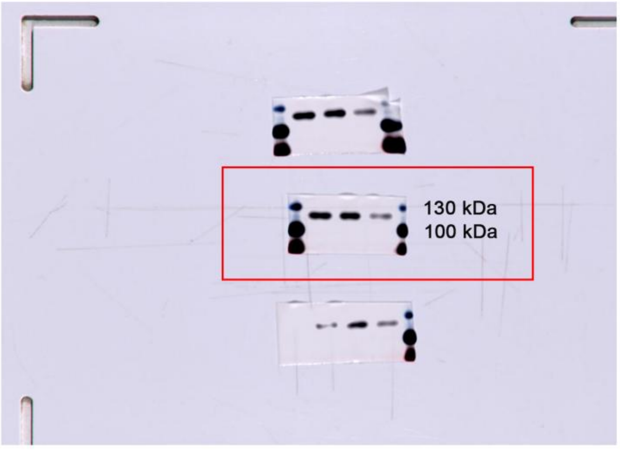

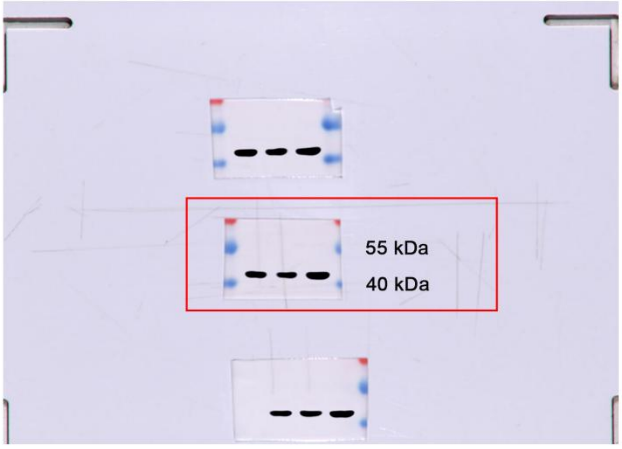

Figure 7

7a

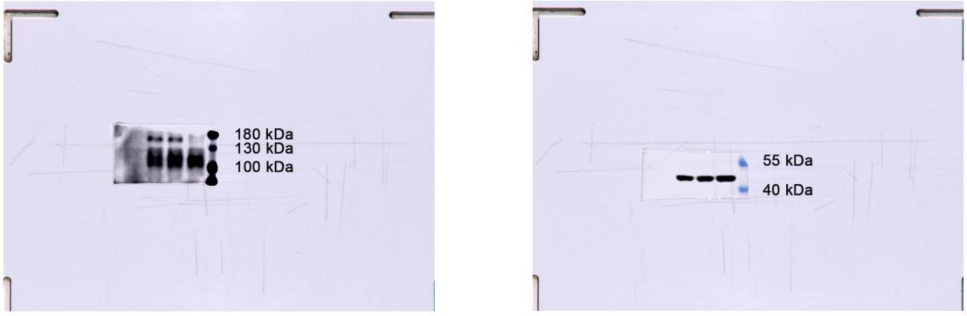

7c

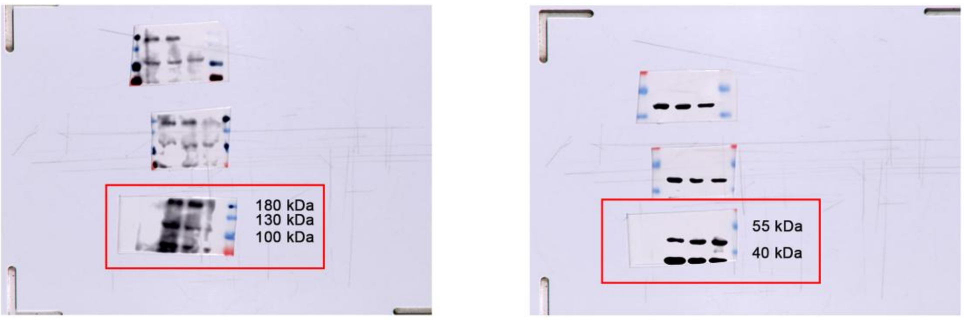

7e

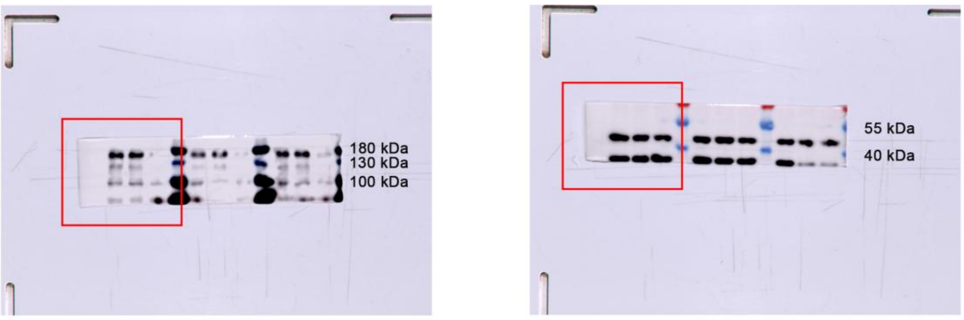

Figure S2

S2a

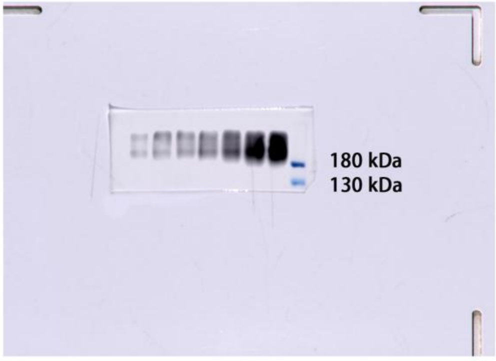

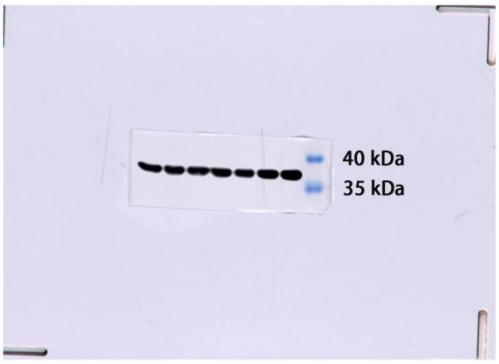

S2c

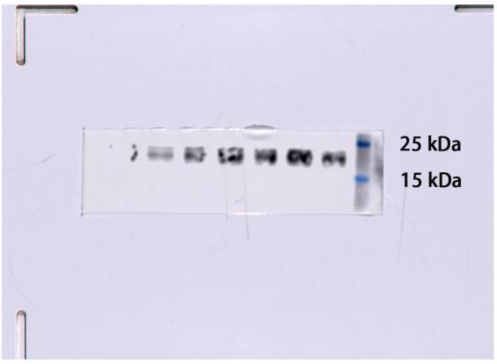

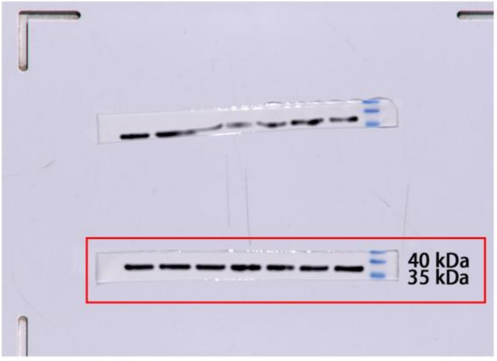

Figure S3

S3a

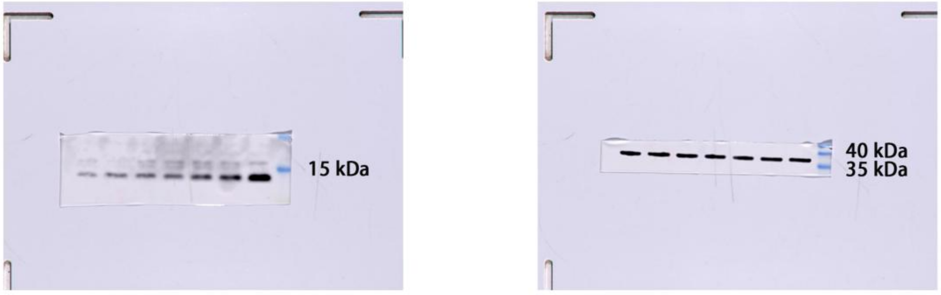

S3c

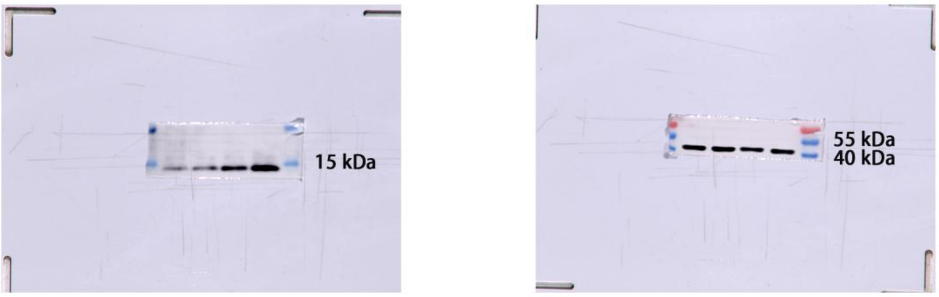

S3e

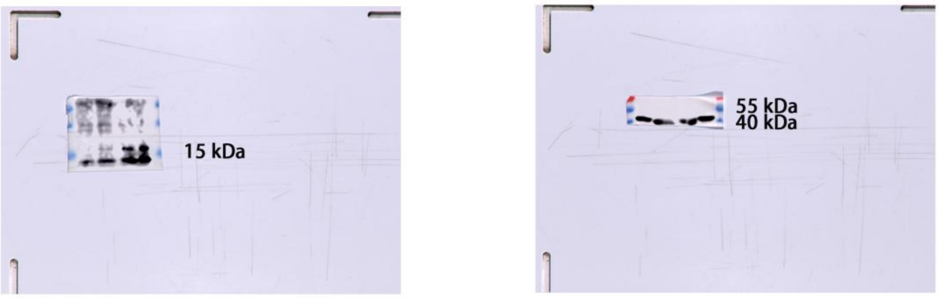

S3j

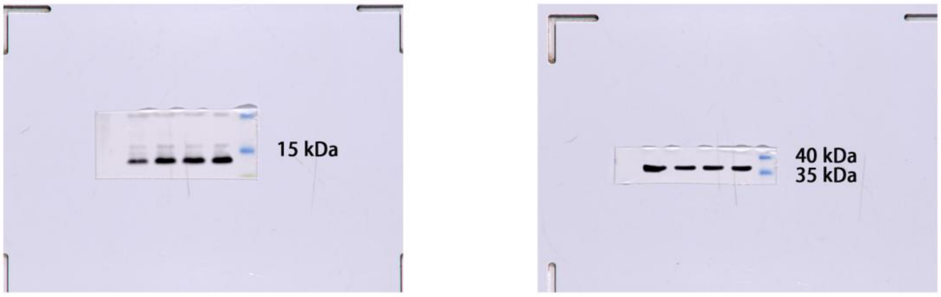

Figure S5

S5a

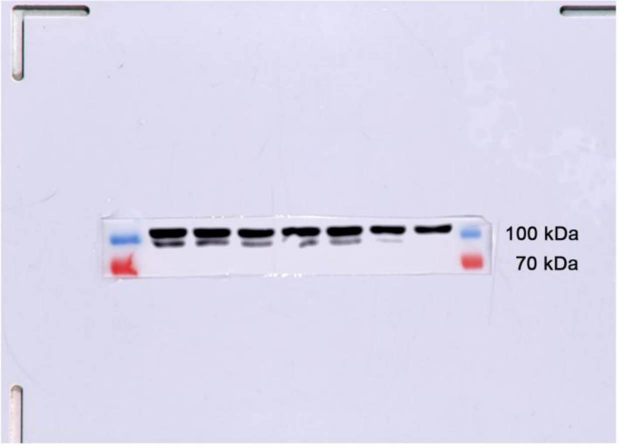

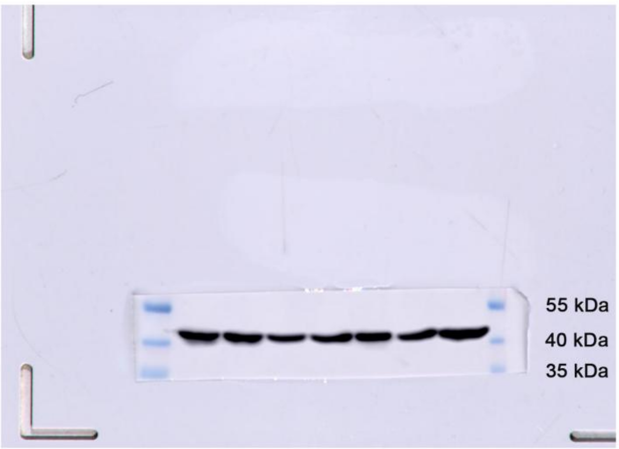

Supplement: Supplementary file 4 — Source Data [file 41467_2024_50711_MOESM4_ESM.zip › Source data/Source data.pdf]
